# A rare case of posterior mediastinal chondrosarcoma: a case report and brief literature review

**DOI:** 10.1093/jscr/rjag060

**Published:** 2026-05-06

**Authors:** Juliana Pardo, Luis Gerardo García-Herreros Hellal, Julio César Granada Camacho, María Alejandra Amaya

**Affiliations:** Universidad de Los Andes, Faculty of Medicine, Cra 1 Nº 18A- 12, Bogotá 111711, Colombia; Fundación Santa Fe de Bogotá, Thoracic Surgery Department, Calle 119 # 7-75, Bogotá 110111, Colombia; Fundación Santa Fe de Bogotá, Thoracic Surgery Department, Calle 119 # 7-75, Bogotá 110111, Colombia; Universidad del Bosque, Faculty of Medicine, Av. Cra. 9 No. 131 A - 02, Bogotá 111321, Colombia

**Keywords:** chondrosarcoma, mediastinal neoplasms, spinal neoplasms, thoracic surgery, video assisted

## Abstract

Chondrosarcomas are malignant tumors originating from cartilage and are rarely found in the posterior mediastinum. Imaging is essential for determining their mediastinal origin. Surgical resection with wide margins is the pillar of treatment due to their aggressive nature and relative resistance to chemo- and radiotherapy. We report a rare case of primary chondrosarcoma of the posterior mediastinum involving vertebrae presenting with neuropathic pain in a 62-year-old woman. We describe an initial minimally invasive surgical approach and a subsequent open approach for margin extension, achieving negative margins. Few cases of posterior mediastinal chondrosarcomas with vertebral involvement have been reported, and it is essential to continue documenting similar experiences to narrow the knowledge gap on these tumors, especially in infrequent locations.

## Introduction

Posterior mediastinal tumors are rare and usually correspond to neurogenic neoplasms, particularly those derived from the neural sheath, such as schwannomas [1]. On the other hand, chondrosarcomas are the second most prevalent bone malignant tumor after osteosarcoma and constitute about 30% of primary thoracic wall tumors [2]. They originate from cartilage, which is why they are more often found in the anterior mediastinum, where this type of tissue is more abundant. Most of them appear in the lower extremities or pelvic bones, and only 10% are found as spinal masses [3]. Surgical resection with wide margins, at least 4 centimeters, is the pillar of treatment, since they have demonstrated a relative resistance to chemo- and radiotherapy. They have an aggressive nature with a high rate of late recurrence and distant metastases [4].

We report a 62-year-old female patient, previously healthy, with a posterior mediastinal chondrosarcoma involving adjacent vertebrae and ribs. We also describe the surgical resection in which we achieved tumor-free margins.

## Case report

**Figure 1:**
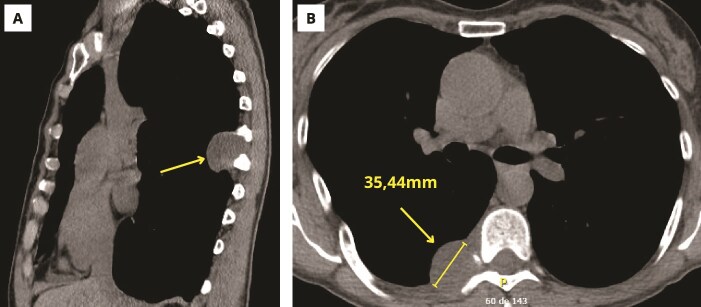
Initial thoracic computed tomography. (A) Sagittal cut. (B) Axial cut.

A 62-year-old female with no relevant medical history was referred to the thoracic surgery outpatient service because of a posterior mediastinal tumor identified on imaging. The patient had been having neuropathic pain for the last few months, with no other symptoms. The thoracic computed tomography showed a mass with a solid and a cystic component of 35 × 44 millimeters, most likely of neural origin, partially eroding the posterior aspect of the eighth rib (Fig. 1). A first surgical approach was conducted in which, through video assisted thoracoscopy, a tumor of approximately 5 centimeters, with malignant appearance, hypervascularization, and a characteristic fish-flesh aspect at the level of the seventh rib and extending through the sixth and eighth intercostal spaces was visualized. The postoperative pathology confirmed a cartilaginous tumor compatible with grade 2 mediastinal chondrosarcoma, with positive expression of S100 protein.

**Figure 2:**
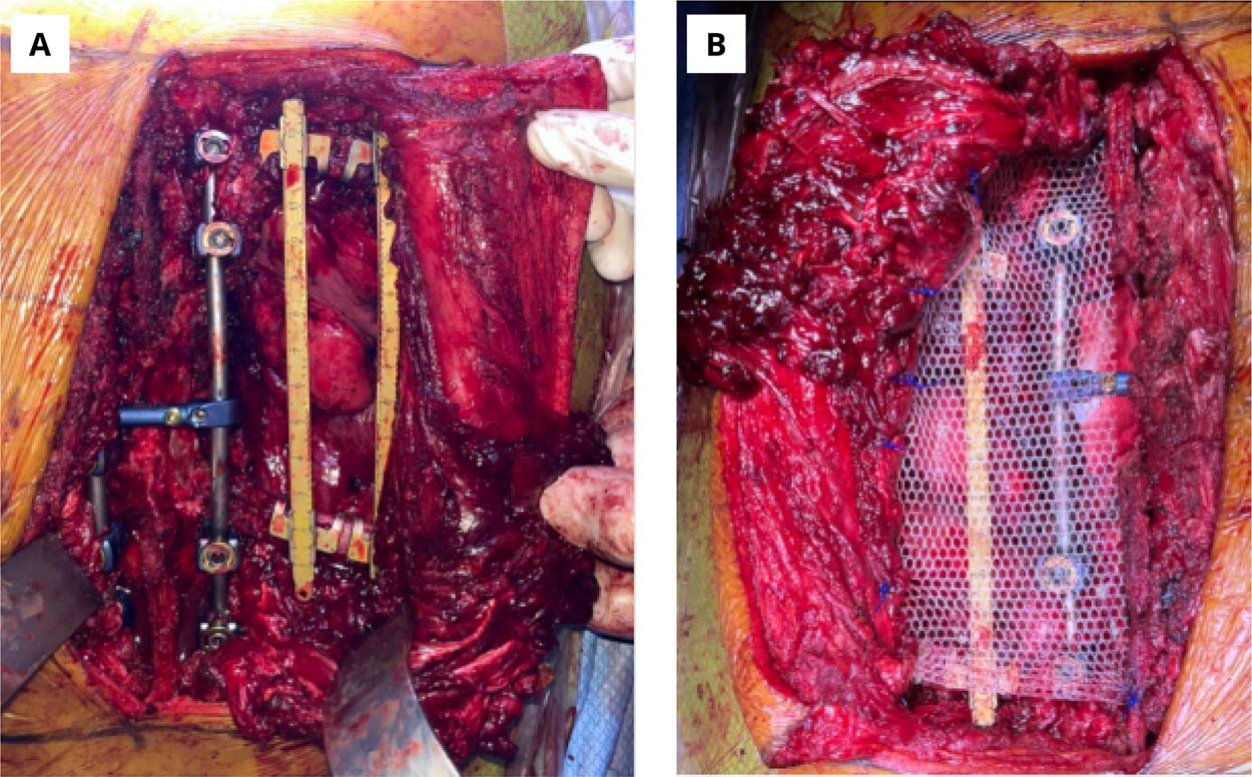
Intraoperative imaging of the thoracic wall reconstruction. (A) Backbone instrumentation and costal arches fixation. (B) Thoracic wall defect repair with prosthetic material.

**Figure 3:**
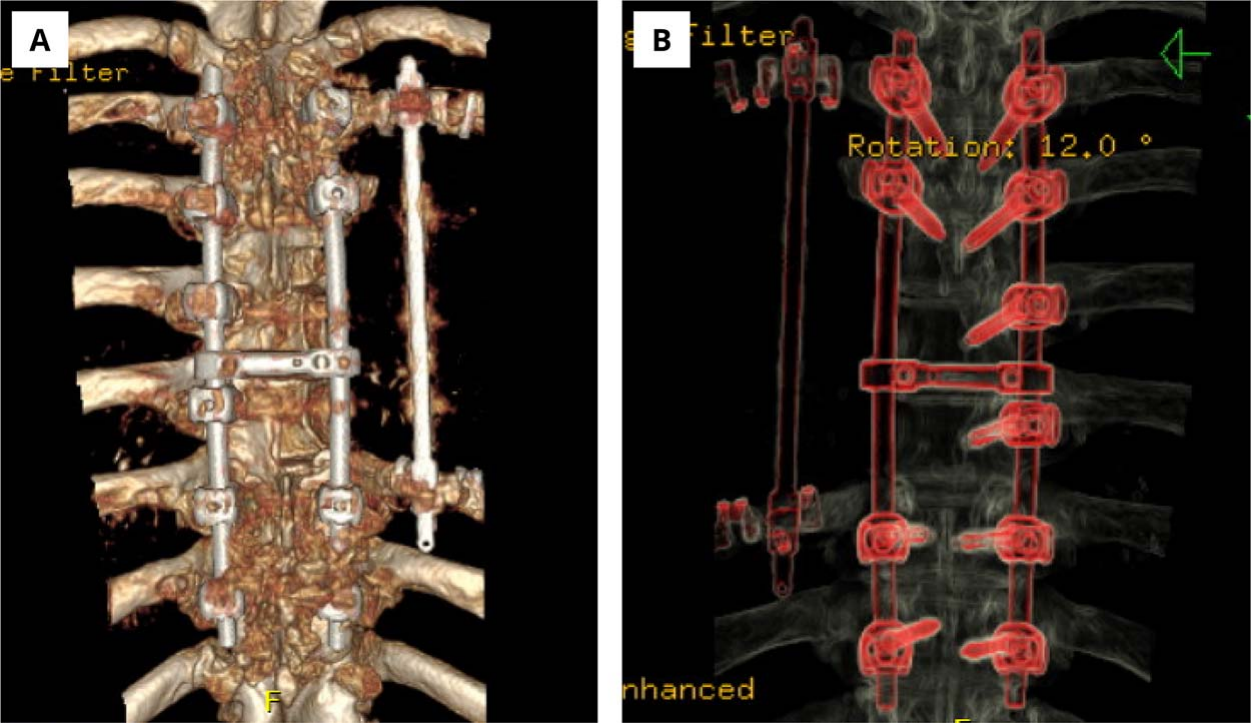
Postoperative computed tomography of the thoracic backbone with three-dimensional reconstruction.

After the histopathological confirmation, the case was discussed in a multidisciplinary medical board along with oncology, radiotherapy and orthopedics. It was determined that the patient would benefit from a new surgical procedure with *en bloc* resection of the tumor mass, margin widening, and thoracic wall reconstruction. The patient was taken to a second procedure with orthopedic and neurosurgery teams. Under general anesthesia in the prone position, the right vertebrocostal sulcus was accessed, flaps were created from the latissimus dorsi muscle, detaching it from its costovertebral junction. The posterior costal arches were exposed from the fifth to the ninth ribs. A wide resection was conducted with neuronavegational guidance. The sixth rib was deperiostized, surrounded, and cut distally to the tumor site. The same procedure was performed for the seventh and eighth ribs. An *en bloc* resection was performed including the lateral end of the corresponding seventh and eight vertebrae and the surgical specimen was extracted. Then, posterior vertebral instrumentation was conducted, and the thoracic wall defect was repaired with a fixation from the fifth to the ninth ribs using two titanium bars in vertical position. Finally, the defect was repaired with prosthetic material (Figs 2 and 3).

The postoperative pathology of the second procedure reported a viable residual chondroid neoplasia with myxoid changes compatible with grade 2 chondrosarcoma, compromising the costal and vertebral fragments as well as the soft intercostal tissues, with a tumor bed of an approximate size of 24 × 15 millimeters. The margins were negative, and the immunohistochemistry again confirmed tumor cell reactivity to the protein S100 marker.

## Discussion

Chondrosarcomas are rare tumors, and even more so in the thoracic wall [2]. There are few cases in the literature reporting lesions involving vertebrae in the posterior mediastinum [5–8]. These tumors tend to grow slowly and be asymptomatic or have nonspecific symptoms, just like our patient who presented with neuropathic pain. On the other hand, the peak incidence is between 30 and 44 years of age and is more common in men [8], which contrasts with our findings. Similarly, chondrosarcomas usually present on diagnostic imaging with calcifications in a rings-and-arcs pattern and a heterogenous content associated with a hyaline cartilage [6]. Nevertheless, in our case, the radiological appearance matched a neural-origin tumor.

Complete surgical resection with wide margins is fundamental and can be complemented with adjuvant radiotherapy [9]. There is only one case reported in the literature describing a similar surgical approach to ours, including posterior thoracotomy, costal resections, partial thoracic vertebral corpectomies, laminectomies, surgical instrumentation, and thoracic wall stabilization, achieving clean margins in pathology and a 9-year follow-up without recurrence [7].

Our report highlights the importance of surgical approach as the main pillar of treatment in chondrosarcomas, considering that it remains the only potentially curative option. It is essential that similar experiences continue to be documented and shared to expand the understanding of this type of tumor, especially in infrequent locations.
